# α-GalCer and iNKT Cell-Based Cancer Immunotherapy: Realizing the Therapeutic Potentials

**DOI:** 10.3389/fimmu.2019.01126

**Published:** 2019-06-06

**Authors:** Yingting Zhang, Ryan Springfield, Siyang Chen, Xin Li, Xiaotian Feng, Rosa Moshirian, Rirong Yang, Weiming Yuan

**Affiliations:** Department of Molecular Microbiology and Immunology, Norris Comprehensive Cancer Center, Keck School of Medicine, University of Southern California, Los Angeles, CA, United States

**Keywords:** iNKT cell, cancer immonotherapy, preclinical modeling, humanized mice, α-GalCer

## Abstract

NKT cells are CD1d-restricted innate-like T cells expressing both T cell receptor and NK cell markers. The major group of NKT cells in both human and mice is the invariant NKT (iNKT) cells and the best-known function of iNKT cells is their potent anti-tumor function in mice. Since its discovery 25 years ago, the prototype ligand of iNKT cells, α-galactosylceramide (α-GalCer) has been used in over 30 anti-tumor clinical trials with mostly suboptimal outcomes. To realize its therapeutic potential, numerous preclinical models have been developed to optimize the scheme and strategies for α-GalCer-based cancer immunotherapies. Nevertheless, since there is no standard protocol for α-GalCer delivery, we reviewed the preclinical studies with a focus on B16 melanoma model in the goal of identifying the best treatment schemes for α-GalCer treatment. We then reviewed the current progress in developing more clinically relevant mouse models for these preclinical studies, most notably the generation of new mouse models with a humanized CD1d/iNKT cell system. With ever-emerging novel iNKT cell ligands, invention of novel α-GalCer delivery strategies and significantly improved preclinical models for optimizing these new strategies, one can be hopeful that the full potential of anti-tumor potential for α-GalCer will be realized in the not too distant future.

Natural Killer T (NKT) cells are CD1d-restricted innate-like T cells expressing both T cell receptor and NK cell markers (1). Invariant NKT (iNKT) cells are the major group of NKT cells in both human and mice. They express the invariant Vα24-Jα18 chains and Vα14-Jα18 TCRα chains in human and mice, respectively (2–4). Since their discovery in the early 1990s, the best-studied function of iNKT cells has been their anti-tumor function. Activated iNKT cells rapidly secrete both Th1 and Th2 cytokines and activate NK and other immune cells to stimulate anti-tumor immune responses (5, 6). The prototypical iNKT cell ligand, α-galactosylceramide (α-GalCer), is a sphingolipid that was first isolated from the marine sponge *Agelas mauritianas* in 1994 by chloroform extraction and HPLC purification techniques (7). Mice injected with free α-GalCer demonstrated potent anti-tumor activity against metastatic B16 melanoma cells (7, 8). Furthermore, α-GalCer demonstrated synergistic anti-tumor effects when co-administered with another chemotherapy agent, adriamycin (8), suggesting α-GalCer has a different target other than the tumor cells themselves. Interestingly, early researchers believed that α-GalCer was a non-specific immunostimulatory agent (8). However, by 1998, studies using knockout mice had concluded that α-GalCer's anti-tumor properties were mediated by CD1d-restricted iNKT cells (9, 10). Several excellent reviews have been published recently on the anti-tumor function of α-GalCer and iNKT cells (5, 6, 11–15). Here we aim to review commonly used preclinical mouse models for α-GalCer and iNKT cell-based cancer immunotherapy to compare and contrast the different approaches in mobilizing iNKT cells for anti-tumor therapies. We specifically focus on the syngeneic mouse B16 melanoma model, a well-established model for human melanoma (16). For a comprehensive review of preclinical modeling of α-GalCer-based cancer therapy targeting diverse tumor types, readers are referred to a recent review by Nair and Dhodapkar (6).

## How Effective is α-GalCer in Anti-Tumor Immunity in Experimental Mice?

Despite extensive literature on the anti-tumor function of α-GalCer and iNKT cells, no standard procedure has been established in delivering the iNKT cell ligands. Procedures vary widely between different research groups. The glycolipid ligands can be administered prior to, simultaneously or after the inoculation of tumor cells at different time points, via intravenous, intraperitoneal or subcutaneous routes, and in free or vehicled forms. Clearly, the scheme, time points, routes and forms for glycolipid deliveries all have a significant impact on the ensuing immune response and therefore the interpretation of the results.

In initial reports on the anti-tumor function of α-GalCer [KRN7000, a close analog of original AGL9b (8)], the glycolipid was injected post B16 cell inoculation. For example, Morita et al. (8) reported that α-GalCer injected 1, 5, and 9 days after subcutaneous inoculation of B16 cells suppressed the tumor volume growth by about 50%. Glycolipid treatment before tumor inoculation represents a prophylactic treatment and may be particularly applicable for future tumor vaccination with specific neoantigens, while the post-tumor inoculation studies are more clinically relevant for anti-cancer therapies.

### α-GalCer Treatment Prior to B16 Melanoma Inoculation

While several reports have demonstrated that pre-treatment with α-GalCer can lead to an anti-tumor response in mice, one study found that injecting α-GalCer immediately before tumor inoculation does not show an anti-tumor effect (17) (Table 1). However, pre-administration of a single-dose α-GalCer 2 days prior to B16 cell inoculation leads to powerful anti-metastatic effect (20). This has been confirmed by ours and other studies (14, 21–23). It is unclear how long the anti-tumor response can last, but it is unlikely to last too long, for example 30 days, as the NKT cells will become anergic by then (17, 25). More kinetic experiments are warranted to determine the duration of this anti-tumor response before the anergy induction because the information will be important for future repetitive administration of α-GalCer and its analogs in clinics.

**Table 1:** Preclinical studies of α-GalCer and iNKT cell-mediated anti-tumor therapies.

**Treatment agent**	**Treatment regime**	**Administration**	**α-GalCer amount/Cell number per mouse**	**Cancer type/mouse model**	**Outcome**	**References**
**Injection of free α-GalCer**						
α-GalCer	Once, immediately before B16 inoculation	Intravenous	2 μg	B16 melanoma	Very little anti-tumor effect	(17)
α-GalCer	Once, shortly after B16 inoculation	Intravenous	100 ng or 500 ng	B16 melanoma	Very little anti-tumor effect	(18, 19)
α-GalCer	Once, simultaneously with B16 inoculation	Intravenous	2 μg	B16 melanoma	Very little anti-tumor effect	(17)
α-GalCer	Once, 2 days prior to B16 inoculation	Intravenous or intraperitoneal	2 μg or 4 nmol	B16 melanoma	Potent anti-tumor effect	(20–23)
α-GalCer	Once, 7 days post B16 inoculation	Intraperitoneal	2 μg	B16 melanoma	Very little anti-tumor effect	(24)
α-GalCer	Multiple, days 0, 4, 8 post B16 inoculation	Intravenous or intraperitoneal	2 or 5 μg	B16 melanoma	Potent anti-tumor effect	(25–28)
α-GalCer	Multiple, from day 3 post B16 inoculation, every other day	Intravenous	2 μg	B16 melanoma	Effective anti-tumor response	(29)
α-GalCer	Multiple, days 1, 5, 9 post B16 inoculation	Intraperitoneal	2 μg	B16 melanoma	Tumor growth inhibition	(9)
α-GalCer	Once and together with anti-PD-1/PD-L1/L2 antibodies	Intraperitoneal	2 μg	B16 melanoma	Enhanced anti-tumor effect, suppressing iNKT cell anergy	(28)
α-GalCer	Once, 7 days post B16 inoculation	Intraperitoneal	2 μg	B16 melanoma/iNOS-KO	Tumor growth inhibition	(24)
α-GalCer	Multiple, every 4 days post B16 inoculation plus ATRA treatment	Intraperitoneal	2 μg	B16 melanoma	Enhanced anti-tumor effect, reducing CD11b^+^ Gr-1^+^ cells	(30)
**Vehicled α-GalCer**						
DC-loaded α-GalCer	Once, simultaneously with B16 inoculation	Intravenous	6 × 10e^5^	B16 melanoma	Enhanced anti-tumor effect, no induction of iNKT cell anergy	(17)
DC-loaded α-GalCer	Multiple, from day 7 post B16 inoculation, every other day	Intravenous	3 × 10e^6^	B16 melanoma	Extended therapeutic window with DC-loaded α-GalCer	(29)
DC-loaded α-GalCer	Multiple, days−7, 14, 21 from tumor cell inoculation	Subcutaneous	6 × 10e^5^	PancO2 pancreatic cancer	Suppressing tumor growth	(31)
DC-loaded α-GalCer	Once, 2 days prior to B16 inoculation	Intravenous	1–3 × 10e^6^	B16 melanoma/hCD1d-KI	Inhibition of B16 metastasis at lower iNKT cell abundance	(21)
B16 loaded α-GalCer	Once, 2 to 4 weeks prior to B16 inoculation	Intravenous	5 × 10e^5^	B16 melanoma	Long-term inhibition of lung metastasis	(32)
B16 loaded α-GalCer	Once, 3 hours post B16 inoculation	Intravenous	3 × 10e^5^	B16 melanoma	Prevention of lung metastasis	(18)
DC-derived exosomes loaded with α-GalCer/OVA	Once or twice, 4 or 4 and 11 days post B16 inoculation	Intravenous	40 μg exosomes	B16.OVA melanoma	Effective suppression of tumor growth, no anergy induction	(19)
Cationic liposomes loaded with α-GalCer	Once, 6 days post B16 inoculation	Intravenous	200 ng liposomes	B16.OVA melanoma	Prolonged survival time	(33)
PLGA nanoparticle encapsulated with α-GalCer/Trp2/gp100	Multiple, days 14 and 7prior to B16 inoculation	Intravenous	5 ng nanoparticle	B16 melanoma	Slowed tumor growth	(34)
PLGA nanoparticle encapsulated with α-GalCer/Trp2/gp100	Multiple, days 5 and 12 post B16 inoculation	Intravenous	5 ng nanoparticle	B16 melanoma	Slowed tumor growth	(34)
α-GalCer loaded to soluble CD1d fused to anti-HER2-svFv	Multiple, every 3-4 days from day 2 post B16 inoculation	Intravenous	40 μg fused sCD1d	B16.HER2 melanoma	Potent anti-tumor effect	(35)
DC-loaded with α-GalCer and B16 cells plus pre-treatment with anti-CD25 Ab	Once, day 7 and anti-CD25 treatment on day 9 prior to B16 inoculation	Intravenous or intraperitoneal	5 × 10e^5^	B16.OVA melanoma	Slowed tumor growth, prolonged survival, depleting Tregs	(36)

### α-GalCer Treatment Post B16 Melanoma Inoculation

Several reports showed that one single injection of α-GalCer either simultaneously or shortly after the B16 melanoma inoculation does not inhibit tumor growth (17–19, 24). Similarly, a single α-GalCer treatment 4 days (19), or seven days after B16 cell inoculation (24) had little beneficial effect on suppressing tumor growth or mouse survival. Therefore, most reports investigating anti-B16 function of α-GalCer have utilized multiple dosages of α-GalCer, typically in a three-dose scheme at days 0, 4, and 8 post B16 inoculation (25–28). In one study, repetitive administration of α-GalCer was initiated at different time points post B16 inoculation (29). α-GalCer was administrated every other day until the end of the experiment on day 14. The free α-GalCer glycolipid demonstrated anti-B16 function as late as 3 days after tumor inoculation, but not beyond 5 days after (29). This may be due to immune-suppression by the established B16 tumors as reported (30). On the other hand, DC-vehicled α-GalCer clearly can extend this treatment window to at least seven days after B16 inoculation (29), suggesting that the vehicled α-GalCer is more efficient in boosting immune response and/or overcoming tumor-led immune suppression.

## Approaches to Improve the Anti-Tumor Efficacy of α-GalCer

Many possible mechanisms have been proposed for the suboptimal efficacies of α-GalCer in anti-tumor clinical trials (5, 6, 11), such as the induction of anergy, the secretion of both Th1 and Th2 cytokines by iNKT cells and immune suppression by the tumors in the microenvironment (30). Many novel α-GalCer analogs have been designed to increase the Th1/Th2 ratio and enhance the anti-tumor immunity (22, 37, 38). While we focus on the anti-tumor function of the prototypic α-GalCer, the chemistry and anti-tumor efficacy and mechanism for these novel α-GalCer analogs have been elegantly reviewed elsewhere (38).

### Approaches to Suppress the Induction of iNKT Cell Anergy

Pioneering work from Fujii and Van Kaer groups demonstrated the induction of long-lasting anergy post α-GalCer activation of iNKT cells (17, 25). The anergy induction not only makes further activation of iNKT cells inefficient, anergic iNKT cells can actually exacerbate tumorigenesis upon further stimulation by glycolipids (25).

The arguably best approach by far to overcome iNKT cell anergy is to load the α-GalCer to dendritic cells (17). Although the absolute amounts of Th1/Th2 cytokines secreted post DC-loaded α-GalCer were not as high as that of free α-GalCer and the cytokines were secreted at a delayed kinetics, the DC-vehicled α-GalCer stimulated higher numbers of cytokine-secreting splenocytes. Importantly, DC-loaded α-GalCer does not lead to iNKT cell anergy (17). More importantly, the DC-vehicled α-GalCer showed more potent anti-tumor activity than free α-GalCer in the B16 melanoma model (17). Interestingly, in this study, both the free α-GalCer and DC-loaded α-GalCer were administered simultaneously with the B16 melanoma cells. While co-injected α-GalCer does not induce immediate anti-tumor activity as discussed above, DC-vehicled α-GalCer can immediately induce anti-tumor activity. Free α-GalCer takes 2 days to induce an anti-tumor response in mice (20), suggesting that these two approaches boosted different downstream effectors. It is particularly important to note that NK cells are only responsible for approximately half of the anti-tumor effect for DC-vehicled α-GalCer (17), while they account for almost all of free α-GalCer mediated anti-tumor function (26, 39, 40). Given the fact that DC-loaded α-GalCer has been widely used in anti-tumor clinical trials (31, 41–43), it is important to further delineate the exact anti-tumor mechanism of DC-vehicled α-GalCer.

The second reported approach to suppress NKT cell anergy is to use exosomes loaded with α-GalCer (19). While in early clinical trials, exosomes loaded with tumor antigens have mostly been tolerated and had little immunostimulatory effects (44, 45), exosomes loaded with α-GalCer as an immune-stimulatory adjuvant led to an effective anti-tumor responses in mice (19). Using a subcutaneous B16 melanoma model, Gehrmann et al. (19) demonstrated that dendritic cells-derived exosomes loaded with α-GalCer administered 4 days after tumor inoculation could effectively suppress tumor growth and extend mouse survival. More importantly, a second injection of loaded exosomes 1 week after the first one can further inhibit tumor growth, suggesting that the first injection with α-GalCer-loaded exosomes did not induce anergy.

Rejuvenating anergic NKT cells at molecular levels is the third approach for suppressing NKT cell anergy. Expression of inhibitory co-stimulatory molecules including PD-1 and PD-L1/L2 is partially responsible for the anergy of NKT cells (28). Three injections of anti-PD-L1/L2 or anti-PD-1 antibodies post α-GalCer activation of iNKT cells could maintain the iNKT cells response for at least 30 days after the α-GalCer treatment (28). This allowed the recovery of iNKT cells to a responsive state and repeated activation of iNKT cells with α-GalCer extended the anti-B16 metastatic function (28). Considering the recent success of anti-PD-L1/2 and anti-PD-1 antibodies in rejuvenating tumor-specific T cells in clinics, future combination treatment with these antibodies and α-GalCer may synergize their anti-tumor functions.

IL-2 has shown anti-anergy function to iNKT cells. In light of its function in breaking anergy of conventional T cells (46), Parekh et al. (25) demonstrated that IL-2, but not IL-12, IFN-γ or IL-4 could re-stimulate the anergic iNKT cells to proliferate both *in vitro* and *in vivo*.

### Additional Approaches to Enhance the Anti-tumor Efficacy by α-GalCer

#### Vaccination With Tumor Cells or Tumor Antigens Complexed With α-GalCer

One major innovation in the field pioneered by the Fujii group is to load α-GalCer to the tumor cells for immunization (18, 32). Even for low immunogenicity tumor cells including B16 melanoma cells, one single vaccination with α-GalCer-loaded tumor cells could stimulate potent tumor-specific CD8^+^ T cell responses. Memory CD4 and CD8 T cells could protect the immunized mice from tumor re-challenge for as long as 6–12 months (32). It was also demonstrated that CD1d expression significantly improved the efficacy of iNKT cell-based therapies, presumably due to increased efficiency of direct killing by iNKT cells. Therefore, CD1d expression on tumor cells can be a positive biomarker for future iNKT cell therapies in clinics, as suggested by another report (47). Importantly, the tumor protection from vaccination in this study is tumor-specific. The mice were only immune to the specific tumor that was used for vaccination (32). On the other hand, α-GalCer-loaded dendritic cells induce short-term tumor resistance against different types of tumors, including melanoma (29), multiple myeloma (48), pancreatic cancer (31) and B cell lymphoma (49). These studies suggested that dendritic cells loaded with α-GalCer induce mostly innate immunity-based non-specific anti-tumor responses including activated NK cells, whereas tumor cells loaded with α-GalCer induce more specific long-term adaptive immunity-based anti-tumor responses.

Several other groups have explored delivering tumor cells or specific tumor antigens with α-GalCer using vehicles such as dendritic cells (36, 50, 51), dendritic cells-derived exosomes (19), dendritic cells loaded with tumor-derived exosomes (52), PLGA nanoparticles (34), cationic liposomes (33), chemically conjugated α-GalCer-tumor peptide antigen compound vaccine (53), or α-GalCer-loaded recombinant soluble CD1d protein fused with single chain antibodies against neoantigen (35, 54). *In vivo*, the tumor antigens are either directly or cross-presented by endogenous dendritic cells to CD8^+^ T cells while the co-delivered α-GalCer is presented to iNKT cells. As expected, all of these approaches have shown enhanced tumor antigen-specific CTL responses and increased IFN-γ secretion in these T cells. These approaches have demonstrated both prophylactic (36), or therapeutic effects (19, 34, 50–53) to challenges by vaccinated tumors. One Phase I trial has been completed using dendritic cells loaded with α-GalCer and the well-established neoantigen NY-ESO-1 (51). It is encouraging that there were increases in NKT cell proliferation, NKT cell-associated cytokine secretion and more importantly, the circulating NY-ESO-1-specific T cells in most (7 out 8) patients (51).

#### More Approaches to Enhance the Anti-tumor Function of α-GalCer

It has been well-established that CD4^+^CD25^+^ T_reg_ cells suppress anti-tumor immunity (55, 56). On the other hand, several reports showed that α-GalCer-activated NKT cells secret IL-2 leading to the expansion of T_reg_ cells (57, 58). Pre-administration of depleting anti-CD25 monoclonal antibody (PC61) 2 days prior to α-GalCer vaccination increased the α-GalCer-induced prophylactic anti-tumor function in a subcutaneous challenge model with B16 melanoma cells (36). However, pre-administration with the same PC61 antibody prior to α-GalCer treatment did not enhance the anti-tumor function of α-GalCer in a therapeutic tumor challenge model with a lung tumor cell line TC1 (59). Interestingly, in the Petersen report (36), α-GalCer challenge and NKT cell activation did not induce an expansion of T_reg_ cells as previously reported (57). This difference is likely due to the different routes of α-GalCer delivery. While in the previous report, delivery of free α-GalCer led to T_reg_ expansion (57), the α-GalCer delivered in dendritic cell-vehicled form in the later study did not (36). More studies are needed to delineate the interaction between iNKT cells and T_regs_ in order to manipulate T_regs_ for the benefit of iNKT cell-mediated cancer therapies.

IFN-γ is one of the major cytokine effectors after α-GalCer administration (1). The high amount of IFN-γ induces immuno-suppressive factors including the iNOS enzyme, which produces nitric oxide and inhibits anti-tumor immunity (60). In iNOS-knockout mice or wild-type mice treated with an iNOS inhibitor, L-NAME, the B16 metastasis was more efficiently suppressed by a suboptimal treatment of α-GalCer (one single treatment seven days after B16 melanoma inoculation) (24). Another study demonstrated that lung metastasis of B16 melanoma was also significantly inhibited by a suboptimal treatment of α-GalCer when the mice were simultaneously treated with all-trans-retinoic acid (ATRA) (30). ATRA, a derivative of vitamin A, can induce the differentiation of CD11b^+^Gr-1^+^ immature myeloid cells and reduce this major nitric oxide-producing population (30).

There are more innovative approaches of enhancing anti-tumor activity of α-GalCer, such as adoptive iNKT cell transfer (61, 62), using artificial antigen-presenting cells to expand iNKT cells *in vitro* (63), co-administration of NK cell activator, IL-18 (64). Altogether, all the reported approaches could increase α-GalCer function. Clearly more research is required to realize their therapeutic potential and achieve the optimal therapeutic efficacy by combining these novel approaches.

## Building Better Mouse Models for Developing α-GalCer-Based Anti-Tumor Therapies

The sharp difference between mouse and human immune systems, including the difference in the CD1d/iNKT cell system, urgently demand better mouse models with improved predictive powers for clinics. In addition to the significantly lower affinities of the human CD1d and iNKT TCR to α-GalCer compared to that of mice (65, 66), human iNKT cells are present at a much lower abundance with very different subset compositions (21, 67, 68). The journey from the original discovery of α-GalCer's anti-tumor function in mice to current clinical trials also suggests that preclinical modeling with more relevant mouse strains is warranted before translating α-GalCer and its analogs into clinics.

One attractive direction to improve the preclinical modeling of α-GalCer-based immunotherapies is to develop mouse models with a human-like CD1d/iNKT TCR system. The first mouse model with a humanized CD1d/NKT cell system is from the Wang group in which human CD1d is expressed under a mouse MHC class I (K^b^) promoter (69). Human CD1d is highly expressed in all nucleated cells as a MHC class I expression pattern. It is not clear how NKT cells are developed in this strain. However, it was clear that the exogenous human CD1d can function as a strong transplantation antigen (69). The second mouse model generated by the Casorati group expressed human CD1d using Lck or CD11c promoters to direct specific human CD1d expression in thymocytes or dendritic cells, respectively (70). By breeding to CD1d-knockout mice, the authors demonstrated that thymocyte-specific expression of human CD1d alone is sufficient to support iNKT cell development (70). However, because of no human CD1d expression on dendritic cells in these pLck-hCD1dTg mice, *in vivo* α-GalCer treatment is not feasible to test the α-GalCer-based cancer immunotherapy. For a human-like iNKT cell population, the Casorati group generated a pre-arranged human invariant Vα24-Jα18 TCRα chain and expressed it as a transgene under the human CD2 promoter (71). In the Jα18-knockout background, which eliminates the expression of mouse iNKT TCRα chain (Vα14), the human Vα24-Jα18 TCRα chain could support the development of human-like Vα24 iNKT cells. The Gumperz group has utilized the humanized SCID mice to generate mice with a humanized CD1d/iNKT cell system. Immune-deficient mice were engrafted with human fetal thymus, liver and CD34^+^ hematopoietic cells. Four surface CD1 gene family members, CD1a, CD1b, CD1c, and CD1d were all expressed *in vivo*. Furthermore, T cell responses have been detected for all the CD1 family members. In addition, α-GalCer can stimulate IFN-γ secretion in the mouse serum, suggesting the NKT cells are developed and functional *in vivo* (72). Nevertheless, more investigation on the immune cell development and adaptive immune responses may be needed before this engrafted system can be widely used for modeling NKT cell-based cancer immunotherapies.

Our group has been working on yet another approach to humanize the CD1d/iNKT cell system. By homologous recombination, we generated a human CD1d knock-in mouse, in which human CD1d is under the endogenous mouse CD1d promoter (21). Consistent with the previous report (70), thymic expression of human CD1d supports NKT cell development. Importantly, this new human CD1d-knock in mouse possesses an iNKT cell population with human-like abundance and similar subset composition in terms of co-receptor expression pattern (21), making this strain a particularly useful tool for modeling *in vivo* human iNKT cell responses to α-GalCer or its analogs. By expressing the pre-arranged human Vα24/Jα18 TCRα chain (23), this further improved mouse strain can be particularly instrumental to test and optimize the glycolipid ligands for anti-tumor therapies. However, since the human Vα24/Jα18 TCRα is a transgene, the current mouse strain is not optimal for investigating the antigen-specific T cell responses during anti-tumor immunotherapies. Nevertheless, since the NK cells and other innate immune cells are not affected by the transgene, this strain can still be used to investigate the innate immunity-mediated anti-tumor function of α-GalCer. To further improve this model, future “knock-in” of human Vα24/Jα18 and Vβ11 genomic regions will be necessary. The continuous improvement of current gene-editing techniques, including CRISPR-Cas9 (73), may make the knock-in more feasible. For preclinical modeling of α-GalCer-mediated anti-tumor therapy, we have demonstrated that prophylactic treatment with α-GalCer in the two CD1d-humanized mouse strains can suppress B16 metastasis (21, 23). Nevertheless, it will be most interesting to investigate whether α-GalCer can suppress B16 melanoma in these humanized mice under therapeutic settings, and if not, how the treatment regimes can be improved for an optimal anti-tumor effect.

In summary, joint efforts from researchers in chemistry, pharmaceutics and immunology fields will bring about more potent α-GalCer analogs, optimized delivery and treatment schemes and much-improved preclinical models. We envision that the α-GalCer-based cancer immunotherapy will be reaching its full potential in clinics in the near future.

## Author Contributions

WY, YZ, RS, and SC participated in conceptualization and drafting of the article as well as critical revision of the article for important intellectual content. All authors participated in writing and revision of the manuscript and gave final approval of the submitted publication.

### Conflict of Interest Statement

The authors declare that the research was conducted in the absence of any commercial or financial relationships that could be construed as a potential conflict of interest.
